# Untangling the Modern Treatment Paradigm for Unruptured Brain Arteriovenous Malformations

**DOI:** 10.3390/jpm12060904

**Published:** 2022-05-30

**Authors:** Brent C. Morel, Blake Wittenberg, Jessa E. Hoffman, David E. Case, Zach Folzenlogen, Christopher Roark, Joshua Seinfeld

**Affiliations:** Department of Neurosurgery, University of Colorado, Aurora, CO 80230, USA; blake.wittenberg@cuanschutz.edu (B.W.); jessa.hoffman@cuanschutz.edu (J.E.H.); david.case@cuanschutz.edu (D.E.C.); zach.folzenlogen@cuanschutz.edu (Z.F.); christopher.roark@cuanschutz.edu (C.R.); joshua.seinfeld@cuanschutz.edu (J.S.)

**Keywords:** brain arteriovenous malformations, ARUBA, unruptured AVM

## Abstract

Brain arteriovenous malformations (AVMs) often present treatment challenges. Patients with unruptured AVMs must consider not only whether they want to be treated, but what treatment modality they would prefer. Vascular neurosurgeons, neurointerventional surgeons, and stereotactic radiosurgeons must in turn guide their patients through the most appropriate treatment course considering the risk of AVM rupture, an individual AVM’s characteristics, and patient preferences. In this review we will look at how the clinical trial “A Randomized Trial of Unruptured Brain Arteriovenous Malformations (ARUBA)” has influenced the approach to unruptured brain AVMs and the treatment modalities available to clinicians to deal with these formidable lesions.

## 1. Epidemiology

Arteriovenous malformations (AVMs) are tangles of dysplastic arteries and veins centered around a nidus that lies within the brain parenchyma [1,2,3,4,5]. Autopsy studies demonstrate unruptured AVM prevalence between 5 and 613 cases per 100,000, with an equally low incidence of 1.10 to 1.42 cases per 100,000 person-years [1,2]. The proliferation of noninvasive neuroimaging, with computed tomography (CT) and magnetic resonance imaging (MRI), has increased the detection of unruptured AVMs. Between 1974 and 1985, incidental AVMs accounted for 13.1% of all AVMs, compared to 45.1% between 2003 and 2017 [6]. The most common presenting symptoms in symptomatic AVMs are intracranial hemorrhage (58%) and the new onset of seizures (34%) [5].

## 2. Rupture Risk

**Figure 1:**
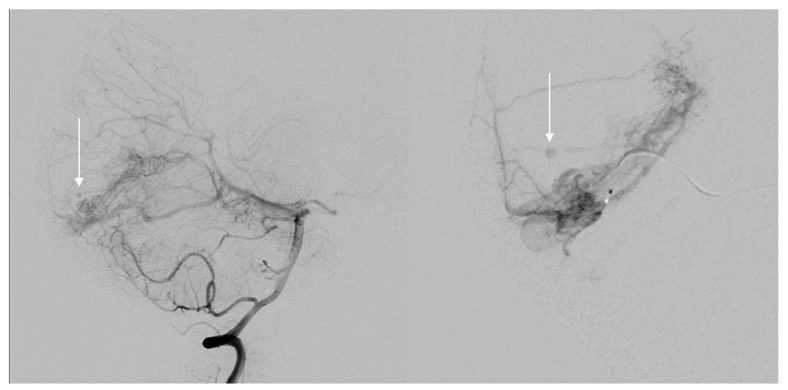
Spetzler–Martin (SM) grade III left occipital arteriovenous malformation (AVM) with supply from the left posterior cerebral artery (PCA) and superior cerebellar artery (SCA) and with an associated perinidal aneurysm (white arrow) that when ruptured is considered a higher risk for being the rupture point.

Although AVMs only carry a 1–4% per year incidence of rupture, resulting morbidity and mortality is severe—with a 30-day mortality rate estimated at 12–67% and permanent neurologic injury in up to 75% of patients [2,3,4]. However, previous rupture raises this risk 2–5 times higher [1,4]. Hemorrhage risk factors include prior hemorrhage, deep venous drainage, fewer draining veins, deep or infratentorial location, a single or few feeding arteries, older patients, female gender, and non-white race. Associated arterial aneurysms (Figure 1), not distinguishing between intranidal and those on proximal vessels, is another hemorrhage risk factor. The 10–20% of AVMs that have any associated aneurysms carry a higher overall hemorrhage rate at 7% per year [1,2,4,5]. 

## 3. The ARUBA Trial

In 2014, the multicenter, randomized controlled trial ARUBA was published. Prior to this, intervention was considered by some to be experimental due to low rupture rates and high procedural morbidity [7]. ARUBA attempted to answer whether preventative treatment of unruptured brain AVMs resulted in better clinical outcomes than medical management alone. This study enrolled 223 adults across 39 sites from 2007–2013 with a mean follow-up time of 33.3 months. It was stopped early after interim analysis showed a 10.1% risk of death or symptomatic stroke in the conservative group versus a 30.7% risk for patients in the interventional group [8]. Five-year follow-up data again showed better outcomes for the conservative management group, which experienced stroke or death in 13.6% of patients versus 35.3% in the intervention arm [9]. 

ARUBA initially seemed to change the landscape of clinical decision making for unruptured brain AVMs. However, soon after the ARUBA trial was published, significant issues were raised regarding the study design and findings. First, only a small number (19%) of patients in the intervention arm underwent microsurgical resection of their AVMs despite microsurgery being first line therapy for Spetzler–Martin (SM) I and II lesions (grade based on size of lesion, deep or superficial venous drainage, and eloquence of surrounding brain parenchyma—with higher grades representing more risky lesions) [10]. Next, other studies found the rates of death, stroke, and neurologic disability in the treatment arm of ARUBA to be significantly higher than expected, with subsequent investigations demonstrating superior treatment outcomes [11,12]. For example, Hong et al. compared the morbidity of AVM therapy in ARUBA with six other studies featuring a total of 956 patients who met ARUBA enrollment criteria. They found the rate of death or symptomatic stroke in interventionally treated patients to be as low as that of the medical arm in ARUBA (8.0% versus 10.1%, respectively) and similar rates of neurologic disability (modified Rankin Score—mRS ≥ 2), with 9.9% in intervened upon patients versus 14.0% in those in the observation arm of ARUBA [11]. In another series, Link et al. performed a retrospective review of their own center’s data for 86 ARUBA-eligible patients from 2004–2017. They reported significantly less stroke or death in their treatment cohort compared to ARUBA (8.3% vs. 30.7%, respectively), with AVM obliteration rates of 92.4% (100% if treatment included microsurgery). Neurologic disability in intervened upon patients was also significantly less at 4.5% in their cohort vs. 46.2% in ARUBA [12]. These series demonstrate disparities among various outcome measures in ARUBA versus similar patient cohorts. Although these series show ARUBA results may not be externally generalized, they are limited by their retrospective nature, therefore prospective data is needed to draw further conclusions. 

Additional critiques of ARUBA include the heterogeneity of patient selection, the lack of standardization of treatment modality in the interventional arm, the relatively short duration of follow up, the high hemorrhage rate compared to existing literature, selection bias in excluding patients with prior hemorrhage or intervention, and not supplying data for associated aneurysm obliteration rates [9,10,11,12,13].

Volovici et al. critiqued ARUBA by examining the trial through the lens of Bayesian analysis. Similar to other criticisms, they argued that as more data became available regarding outcomes for intervention, it became clear that ARUBA was the outlier with its unfavorable outcomes. They posited that the trial suffered from poor decision making for treatment modality in the interventional arm, particularly notable in the underrepresentation of microsurgical resection for SM I and II lesions. Furthermore, they felt that allowing centers to choose their own standard treatment modality was antithetical to the underlying ARUBA hypothesis that treatment was ultimately experimental. Although five-year follow-up data was published for ARUBA, it remains inadequate, as hemorrhage risk spans a lifetime. Poor interventional choice in ARUBA may have resulted in poor AVM obliteration rate and subsequently poor patient outcomes in the interventional arm [14].

More recent investigations have now looked at subgroups of ARUBA and ARUBA-eligible patients to determine which specific AVM patients may benefit the most from intervention. Nerva et al. analyzed their own ARUBA enrollment criteria-eligible patients by stratifying patients based on SM grade. With this stratification of 61 patients, SM I and II AVMs treated with surgery with or without pre-operative embolization all had radiographic cure and mRS of 0–1, indicating good neurologic outcome. They also found less impaired outcomes in the SM I and II group compared to SM III-V [15]. Stefani et al. found no association in the ARUBA trial between risk of stroke or death and the SM grade of the AVM in the medical arm, prompting a call for further study into which unruptured AVMs present the highest risk of hemorrhage [16].

It has been suggested that although ARUBA highlighted the risk of intervention, its conclusion that medical management is superior may not hold true over time. Treatment of patients at high-volume, multidisciplinary medical centers with good patient selection is key to successful treatment and favorable outcomes [17]. 

Despite the significance of the findings from ARUBA, and the controversy surrounding them, its overall effect on practitioners’ approaches to the management of unruptured AVMs has been diminutive. An examination of the Nationwide Readmission Database revealed no significant difference between treatment of unruptured AVMs in the United States before and after ARUBA (8.0–9.2 per 10 million AVM interventions before ARUBA versus 7.7–8.3 per 10 million after ARUBA) [18]. Similarly, Sussman et al. also showed no significant change in unruptured AVM case volume before and after ARUBA [19].

## 4. Treatment Options

Determining when and how incidentally discovered AVMs should be intervened upon remains a widely debated topic. Given the high stakes should an AVM rupture, a detailed discussion regarding natural history and management strategies is essential following diagnosis. Treatment options include conservative management through watchful waiting, microsurgical resection, catheter-directed embolization, stereotactic radiosurgery (SRS), or a combination of the above. Prior and ongoing studies have aimed to determine not only which patients are appropriate for intervention, but also the risk–benefit profile of these modalities. Naylor et al. propose that if the patient does not have limiting old age or medical comorbidities, then their lifetime risk of rupture is higher than the risk of treatment and therefore patients should undergo treatment [6]. However, the balance of lifetime rupture risk compared to treatment risk has not been fully elucidated. 

Treatment success varies considerably. In a review of 1809 patients from 11 studies on AVM obliteration rates, using any treatment modality for SM grade I or II AVMs, Talaat et al. showed complete obliteration rates ranging from 36.5–100%. Symptomatic procedure-related hemorrhage rates were between 0% and 7.3% [20]. When each treatment modality is analyzed individually, Liu et al. in a review of 28 studies with an aggregate 5852 patients demonstrated obliteration rates of 98% for microsurgery, 87% for embolization, and 68% for SRS. They also reported complications of 1% stroke or death for microsurgery, 4% for embolization, and 8% for SRS [21]. 

### 4.1. Microsurgery

**Figure 2:**
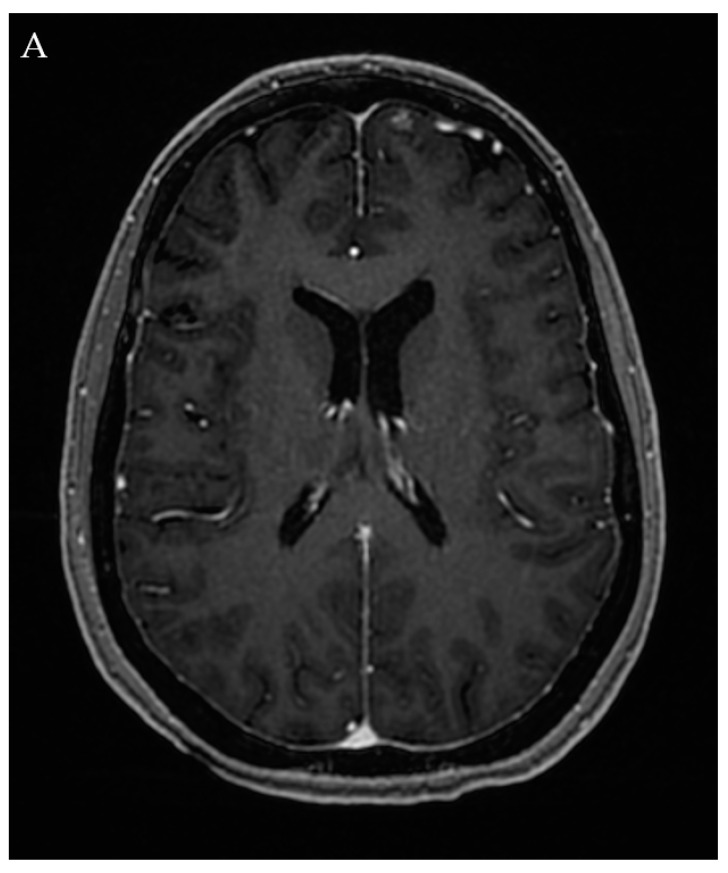

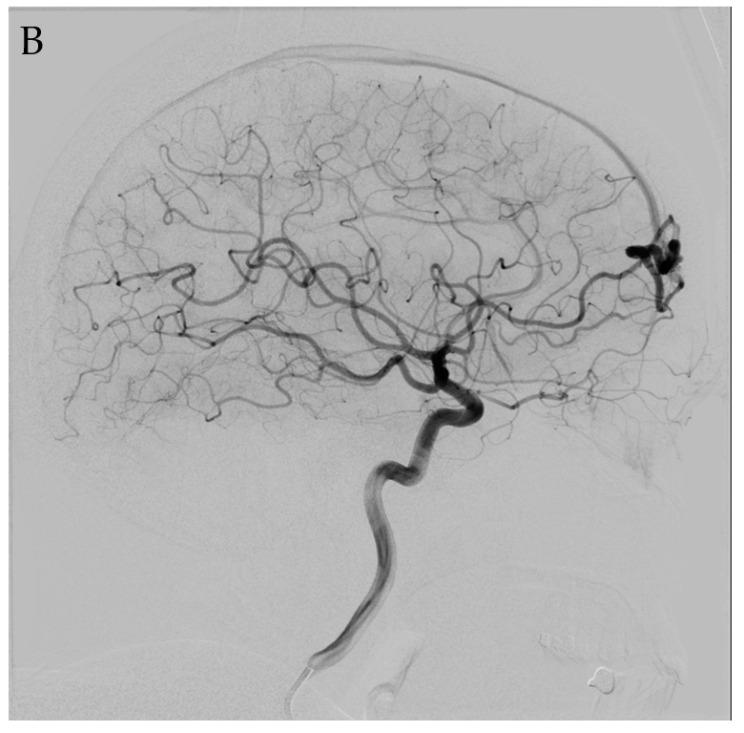

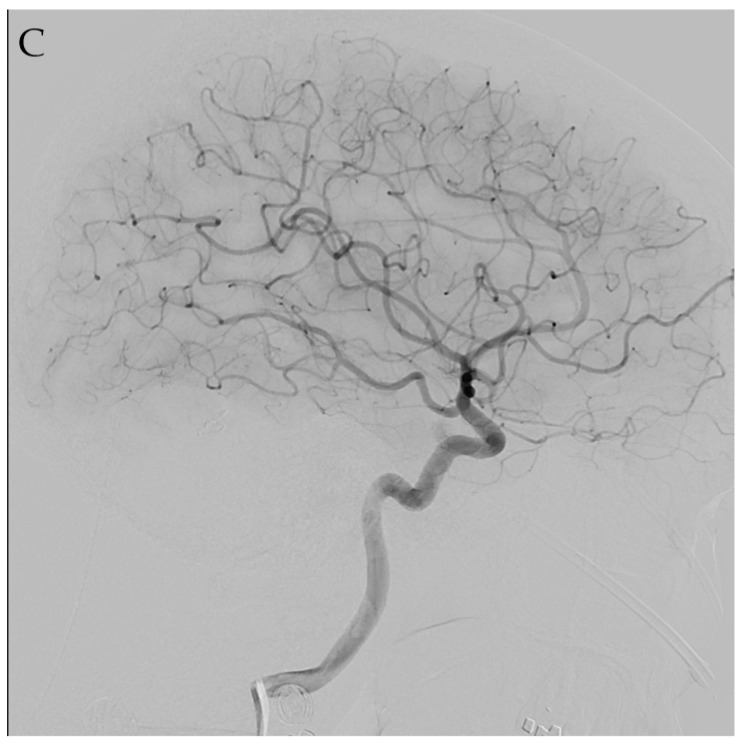
A 47-year-old female who presented with headaches and dizziness found to have an unruptured left anterior frontal lobe SM grade I AVM on MRI (**A**) with arterial supply from an orbitofrontal branch of the anterior cerebral artery (ACA) with venous drainage via a cortical vein into the anterior superior sagittal sinus (**B**). The patient was presented with microsurgical resection, embolization, and SRS as treatment options. Given the risk of residual AVM after embolization and a latent period between SRS and AVM obliteration, the patient opted for microsurgical resection. Follow-up angiography demonstrated no residual lesion (**C**).

Microsurgical resection has been shown to be very effective in low SM grade AVMs, but has unacceptable morbidity with increasing AVM size, deep venous drainage, or eloquent location (Figure 2) [1,2,4,22]. Lawton et al. showed that out of 232 AVM surgeries on SM grade I and II AVMs, complete resection was able to be obtained in 100%, with 97% of patients having an unchanged or improved neurologic baseline following treatment. They compare these rates to those of their review of 1297 patients with mostly low-grade AVMs who underwent embolization alone with a lower cure rate of 29%, with a 6.2% broadly defined morbidity and a 1.6% mortality rate [23]. 

### 4.2. Endovascular Embolization

Endovascular techniques, most commonly using liquid embolic agents such as Onyx (Medtronic, Minneapolis, MN, USA) and n-butyl-2-cyanoacrylate (n-BCA; Trufill, Cordis Neurovascular, Miami Lakes, FL, USA), are often employed as an adjunct prior to microsurgery to reduce intraoperative bleeding or prior to SRS to shrink the AVM nidus to a suitable size. Surgery with pre-operative embolization engenders shorter operative time and less blood loss, but with no difference in complication or obliteration rates [24]. Embolization has also been increasingly studied as a solo technique both for palliation by reducing venous hypertension or to cure small AVMs with one feeding artery and a sufficiently large pedicle diameter [1,2,4,22].

Endovascular techniques alone can also provide a minimally invasive instantaneous cure. In a review of 15 studies looking at 597 patients undergoing embolization comprised of 70.9% SM I-III AVMs, Wu et al. showed complete obliteration in 58.3% of patients, but with 24.1% of patients experiencing clinical complications (9.7% of which were hemorrhage) and procedural mortality of 1.5%. Procedural complications included vessel perforation, embolysate extravasation, catheter disconnection, catheter or guidewire breaking, trapped/glued/retained catheter tip, and stroke. Pre-procedure embolization eliminated high-risk features associated with surgery or shrunk the AVM down to a size amenable to SRS. This review identified features favorable for embolization, including a small nidus size of 1–3 cm, feeders from a single arterial pedicle or at least from a single vascular territory, superficial or large arterial feeders, visualization of venous drainage, room for 2–3 cm of reflux, location in non-eloquent brain tissue, and a low SM grade. In fact, in SM grade I-III AVMS selected for their favorable anatomical characteristics, endovascular embolization alone can achieve over a 90% obliteration rate [25]. 

Baharvahdat et al. examined endovascular treatment of 224 SM grade I and II lesions and showed complete obliteration in 92% (62.1% of which were in one session) with 0.4% mortality and 5% permanent neurologic deficit [26]. The same group also looked at specifically SM III lesions treated with embolization and found among 65 patients an 87.7% cure rate. However, complication rates were high, with a 20% hemorrhage rate, 7.7% stroke rate, 6.2% rate of neurologic deficit (15.4% having mRS 3–5), and a 3% mortality rate. They again note that the high cure rate should make embolization a consideration for cure in SM III lesions, given their variable size, location, and venous drainage, if other options are also considered high risk [27]. These studies also point out the high efficacy of embolization in appropriately selected low-grade AVMs. 

### 4.3. Stereotactic Radiosurgery

**Figure 3:**
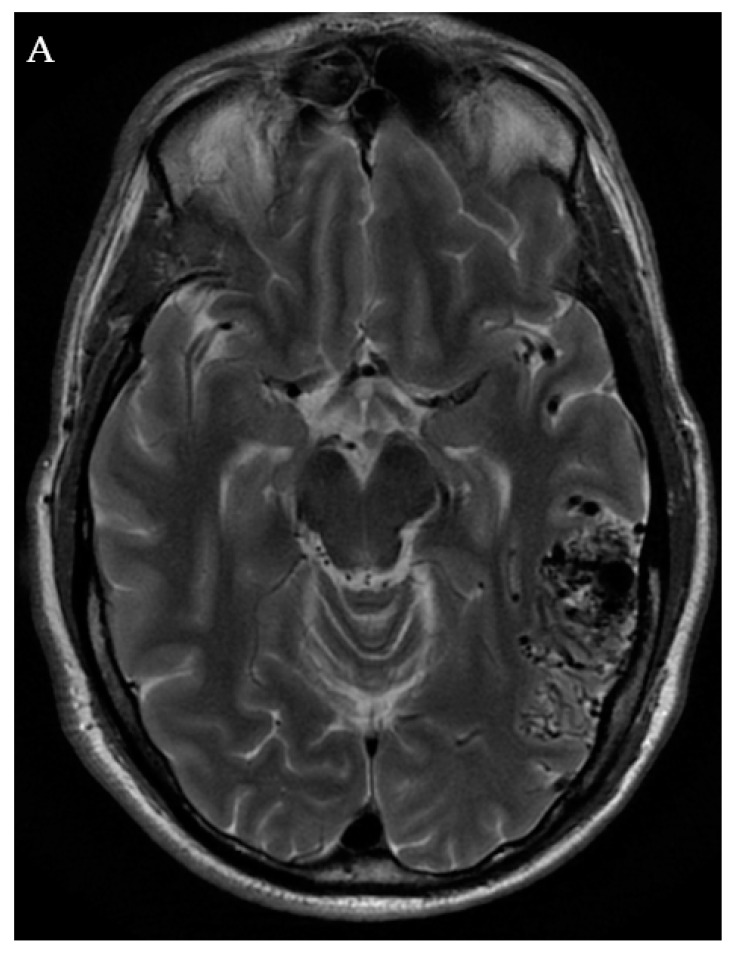

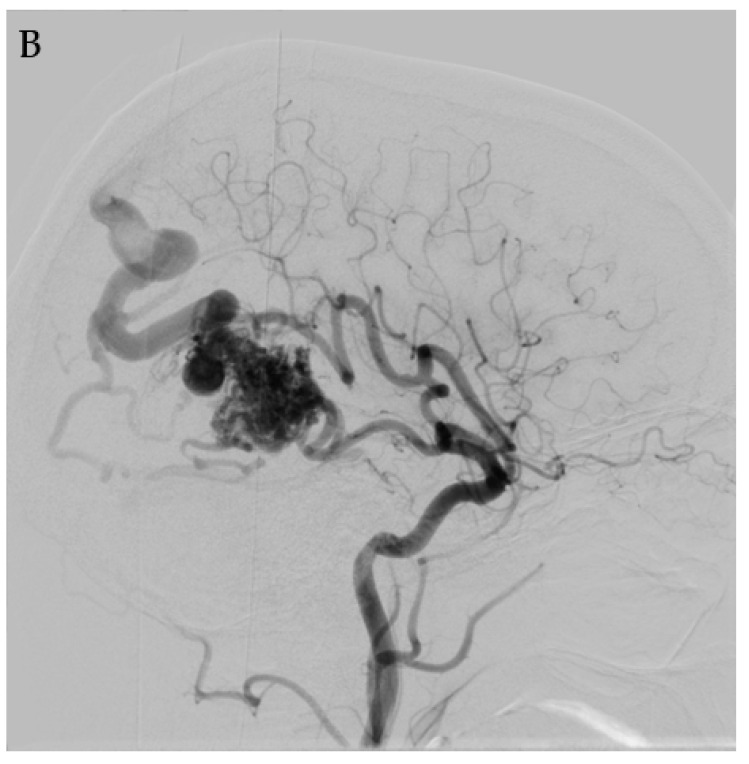

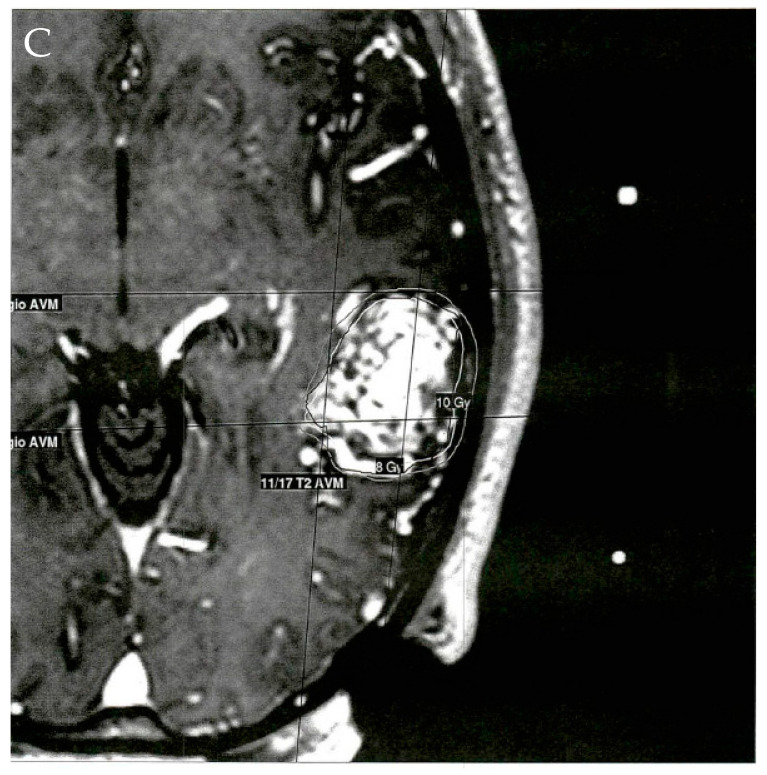

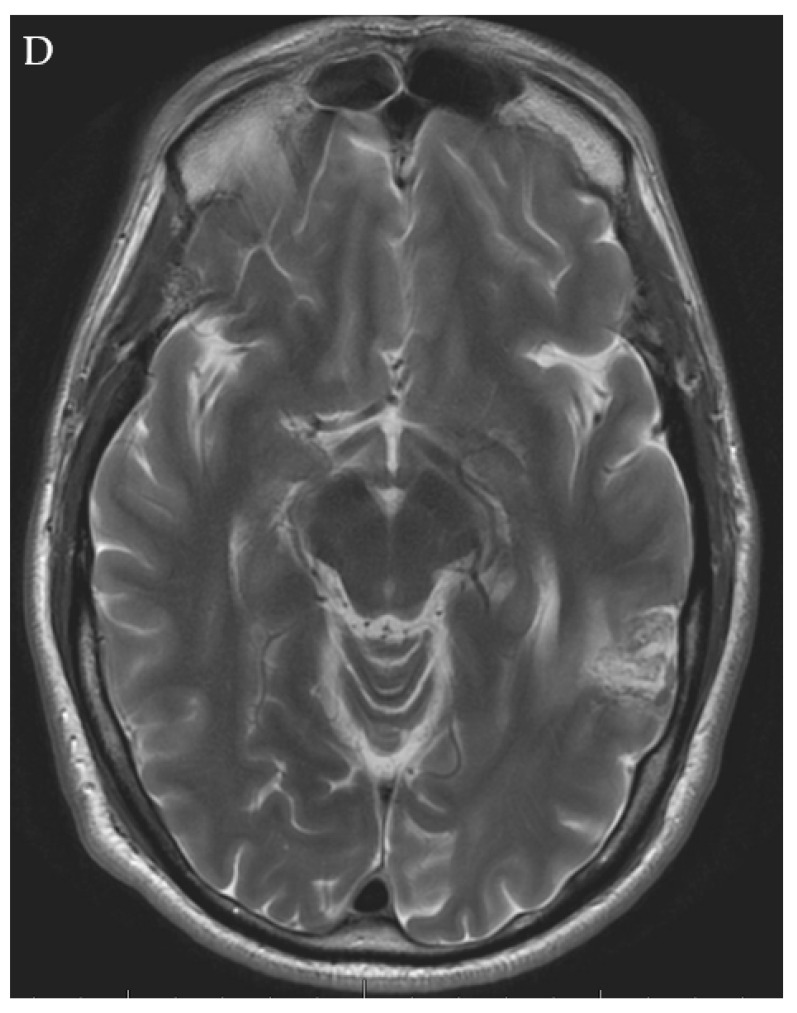

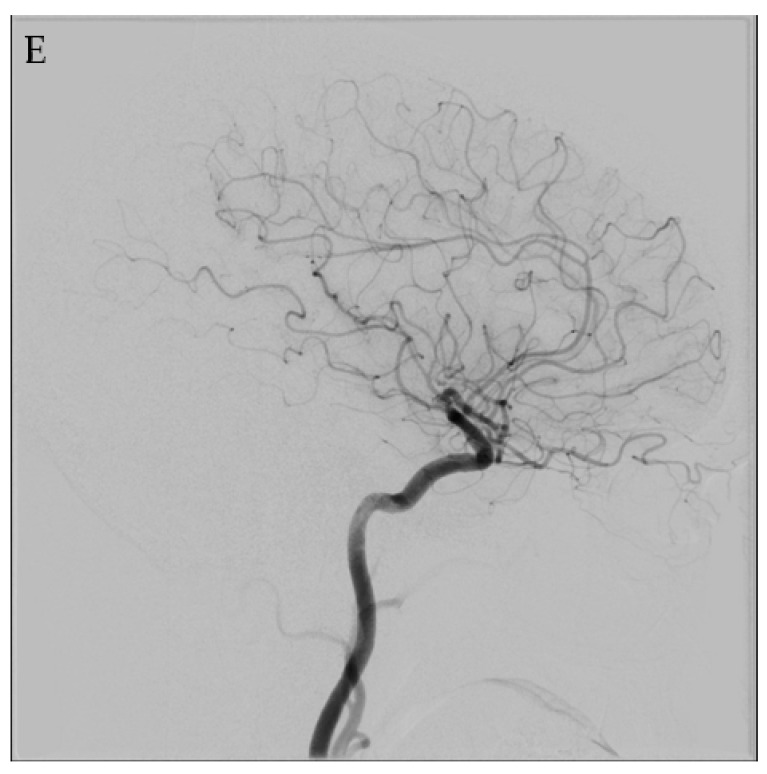
A 48-year-old male who presented with a seizure found to have an unruptured left posterior temporal lobe SM grade II AVM with arterial supply from the left middle cerebral artery (MCA), PCA, and left middle meningeal artery (MMA) (**A**,**B**). Given the location of the lesion, a WADA test was performed, which demonstrated left brain language dominance. The patient was a professional musician, therefore he wanted to minimize the risk of peri-procedural deficits. As a result of the eloquent location of the lesion, the patient underwent a dose-staged SRS plan over three treatment sessions at 0, 4, and 10 months (**C**). Dose-staged SRS was felt by the treating physician to minimize risk of deficit. Follow-up imaging demonstrated complete resolution of the AVM two and a half years after initiation of SRS (**D**,**E**). This case illustrates the importance of personalized patient consideration and preference.

SRS can be used in lesions in which surgical risk is too high and can achieve up to a 60–80% obliteration rate after 3–5 years, but the patient must accept a risk of hemorrhage during the lag time to cure [1,2,4,22]. Risks include radiation necrosis, edema, and a delay post treatment time of 2–4 years prior to cure of the lesion [4]. SRS is typically limited by AVM size, with obliteration rates dropping precipitously for lesions greater than 3 cm in diameter [28,29,30]. Staging SRS treatment, either by splitting the target into two or more smaller volumes or performing the treatment in multiple lower dose fractions, can enable treatment of larger AVM niduses while maintaining adequate efficacy and safety profiles (Figure 3) [31]. Alternatively, embolization prior to SRS can be helpful to decrease target volume (Figure 3). Chen et al. refuted the notion that embolic material can reduce efficacy of SRS in their study of 106 patients split into two equal cohorts of SRS with or without upfront Onyx (Medtronic, Minneapolis, MN, USA) embolization, showing no difference between obliteration rates [28]. Further study is needed to determine whether pre-SRS embolization definitively improves outcomes.

### 4.4. Multimodality Therapy

**Figure 4:**
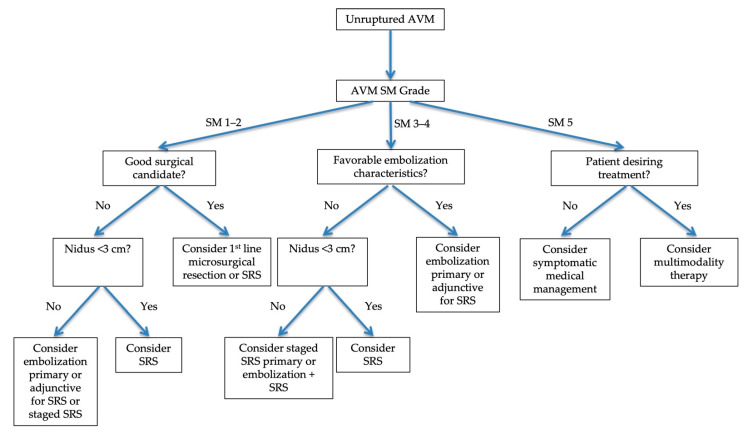
Flowchart outlining treatment modality decision-making algorithm for unruptured AVMs. Decision making is complex and involves consideration of AVM characteristics and shared decision making with the patient.

Multimodality treatment can raise overall obliteration rates, especially in complex high-grade lesions. In their case series of 265 patients who underwent interventional treatment, Nataraj et al. demonstrated an AVM obliteration rate of up to 92% in SM I-IV AVMs and 53% in SM V lesions. Their series included 28% SM III, 19% SM IV, and 14% SM V lesions. Specifically, when applying multiple treatment modalities, they had a 99% cure rate when combining embolization with microsurgical resection, a 70.5% cure rate when combining embolization with SRS, a 100% cure rate when combining microsurgical resection with SRS, and a 100% cure rate when combining all three treatment modalities. Of the 14 patients who received all three modalities, 93% had favorable outcomes as measured by the Glasgow Outcome Scale. Incorporating surgery as one of the combined treatments improved the chance of good recovery. The authors note that they intended treatment in 96% of SM III lesions, 94% of SM IV lesions, and 75% of SM V lesions. Their goal was the complete obliteration of all treated lesions. They note a bias towards embolization as an adjunctive treatment to enable SRS or microsurgical resection [32]. This case series shows that experienced providers can approach lesions that were previously considered too dangerous to treat. By utilizing combined therapies at the discretion of the treating provider based on lesion characteristics and patient preference, reasonable success can be expected, although it is still important to be mindful of the escalating risk of treatment as the SM grade increases. Although AVM treatments involve nuanced decision making, a basic algorithm is presented in Figure 4.

## 5. Conclusions

Unruptured brain AVMs are rare, but are associated with high morbidity and mortality. Neurosurgeons, neurointerventionalists, and radiosurgeons are becoming increasingly sophisticated in the treatment of these lesions driven by patient selection supported by a growing body of evidence taking into account demographics and the angioarchitecture of these lesions. While ARUBA did not definitively conclude the best treatment choice for unruptured AVMs or significantly change practice patterns, it did prompt further investigations to help refine which lesions should be managed conservatively versus treated. Data support relatively safe and highly effective treatment of SM I and II lesions with microsurgical resection, although poor surgical candidates can reasonably consider embolization if the AVM bears appropriate anatomical features or SRS if they wish to forgo a major procedure and can tolerate obliteration lag time. Higher grade lesions carry increased risk with procedures, beckoning the question of whether to proceed at all, but data show viable treatment options with modality preference based on the characteristics of the lesion. Ultimately, conversations between the patient and their doctor must drive the decision of whether to treat at all, and if so, which modality to choose.
